# Nano-Hydroxyapatite vs. Xenografts: Synthesis, Characterization, and In Vitro Behavior

**DOI:** 10.3390/nano11092289

**Published:** 2021-09-02

**Authors:** Cristina Rodica Dumitrescu, Ionela Andreea Neacsu, Vasile Adrian Surdu, Adrian Ionut Nicoara, Florin Iordache, Roxana Trusca, Lucian Toma Ciocan, Anton Ficai, Ecaterina Andronescu

**Affiliations:** 1 Department of Science and Engineering of Oxide Materials and Nanomaterials, Faculty of Applied Chemistry and Materials Science, University Politehnica of Bucharest, 060042 Bucharest, Romania; cristinadumitrescu0@gmail.com (C.R.D.); adrian.surdu@upb.ro (V.A.S.); adrian.nicoara@upb.ro (A.I.N.); anton.ficai@upb.ro (A.F.); ecaterina.andronescu@upb.ro (E.A.); 2 National Research Center for Micro and Nanomaterials, Faculty of Applied Chemistry and Materials Science, University Politehnica of Bucharest, 060042 Bucharest, Romania; truscaroxana@yahoo.com; 3 Department of Biochemistry, Faculty of Veterinary Medicine, University of Agronomic Science and Veterinary Medicine, 011464 Bucharest, Romania; floriniordache84@yahoo.com; 4 Prosthetics Technology and Dental Materials Department, Carol Davila University of Medicine and Pharmacy, 020022 Bucharest, Romania; tciocan@yahoo.com; 5 National Research Center for Food Safety, University Politehnica of Bucharest, 060042 Bucharest, Romania

**Keywords:** biomaterial, bone substitute, apatite, microwave-assisted hydrothermal synthesis

## Abstract

This research focused on the synthesis of apatite, starting from a natural biogenic calcium source (egg-shells) and its chemical and morpho-structural characterization in comparison with two commercial xenografts used as a bone substitute in dentistry. The synthesis route for the hydroxyapatite powder was the microwave-assisted hydrothermal technique, starting from annealed egg-shells as the precursor for lime and di-base ammonium phosphate as the phosphate precursor. The powders were characterized by Fourier-transform infrared spectroscopy (FTIR), X-ray diffraction (XRD), scanning electron microscopy (SEM), energy-dispersive X-ray analysis (EDAX), transmission electron microscopy (TEM), X-ray fluorescence spectroscopy (XRF), and cytotoxicity assay in contact with amniotic fluid stem cell (AFSC) cultures. Compositional and structural similarities or differences between the powder synthesized from egg-shells (HA1) and the two commercial xenograft powders—Bio-Oss^®^, totally deproteinized cortical bovine bone, and Gen-Os^®^, partially deproteinized porcine bone—were revealed. The HA1 specimen presented a single mineral phase as polycrystalline apatite with a high crystallinity (X_c_ 0.92), a crystallite size of 43.73 nm, preferential growth under the c axes (002) direction, where it mineralizes in bone, a nano-rod particle morphology, and average lengths up to 77.29 nm and diameters up to 21.74 nm. The surface of the HA1 nanoparticles and internal mesopores (mean size of 3.3 ± 1.6 nm), acquired from high-pressure hydrothermal maturation, along with the precursor’s nature, could be responsible for the improved biocompatibility, biomolecule adhesion, and osteoconductive abilities in bone substitute applications. The cytotoxicity assay showed a better AFSC cell viability for HA1 powder than the commercial xenografts did, similar oxidative stress to the control sample, and improved results compared with Gen-Os. The presented preliminary biocompatibility results are promising for bone tissue regeneration applications of HA1, and the study will continue with further tests on osteoblast differentiation and mineralization.

## 1. Introduction

After blood transfusions, bone grafts are placed on the second position as the most frequent tissue transplantation used worldwide [1]. Multiple factors determine the occurrence of alveolar bone defects, but the most common are osseous deficiency, the resection of tumors, alveolar bone loss due to periodontal disease, and subsequent tooth loss [2]. Bone defects require rehabilitation, mainly to avoid severe alveolar bone resorption that compromises bone quantity, morphology, and quality, which prevents the failure of dental implant placement, the maintaining of the normal anatomic outline, the elimination of empty space, aesthetic restoration, as well as drugs encapsulation and delivery [3,4,5]. Until now, only the autografts and, partially, allografts could fulfil the desired properties such as biocompatibility, osteoconductivity, and osteoinductivity for a better bone substitution ability, albeit entailing important risks [6,7,8,9,10]. Xenografts (mammalian bone source) have been used for more than thirty years and are still being used with good clinical results, osteoconductive features, very good biocompatibility, high availability (size and quantity), and low cost, but also involving many disadvantages [3,11,12,13,14,15,16,17,18,19].

Therefore, natural sources for biological apatite synthesis have been extended, year by year, from mammalian bone sources next to fish bones and scales [20,21,22], egg-shells [23,24], and exoskeletons of marine organisms (snails, starfish, coral, and seashell) [25,26,27,28] or botanic sources (Calendula flower, Papaya leaf, and orange peel), where all of them need chemical and thermal preparation before use as a mammalian xenograft. However, the interest in this topic is still present, a fact proved by several recent publications [23,24,25]. Mammalian bone, after thermal treatments, provides so-called biological apatite [3,11,12,13,14,15,16,17,18,19]; egg-shells (hen, crocodile etc.) and marine organism skeletons, including corals and starfish, are special sources of biogenic calcium carbonate [23,24,25,26,27,28]; phytogenic calcium carbonates could be used after the extraction process in the original form, preserving the porous structure, calcium-rich source, and biomolecules (Ovalbumin, Carotene, Papain, and vitamins) [10,11].

The great advantages of alloplastic bone substitute consist of a wide range of compositions, sizes, shapes, textures, synthesis methods, biocompatibility and bioresorbability, high quantities of availability, and cost-effectiveness [29,30,31,32]. Despite these advantages, they still need improvements in order to enhance their integration into the physiological environment, their bioactivity, or biotolerance [33,34,35].

Nowadays, one of the most promising approaches is hydroxyapatite synthesis from hen egg-shell sources due to the mimetic composition and structure of the carbonated apatite obtained compared with human bone [36,37,38]. Such carbonated apatite is prepared using several routes, including dry methods (solid state [39] and mechanochemical synthesis [40]), wet methods (precipitation [41,42,43], hydrolysis, sol–gel [44], emulsion [45], sonochemical [46], hydrothermal [47,48], and solvothermal [49,50]), or high-temperature methods (pyrolysis, combustion, and microwave heating [50]). Each of the abovementioned methods induces different morphologies of micro- and nano-size HA particles: rods [51,52], sheets [53], spheres [54], wires [55], fibers [56], flower, worm, hexagonal prism, platelet, lath, strip, dandelion, chrysanthemum, rosette, or spheres [20]. Among those, the hydrothermal method generates conditions for high crystallinity and stoichiometric hydroxyapatite synthesis (Ca/P ratio around 1.67) from shells of calcium carbonate precursor, usually with a rod-like morphology and hexagonal unit cell symmetry [57,58]. Moreover, combining hydrothermal synthesis (HT) with microwave thermal treatment (MW) was observed to lead to a higher crystal size of HA from egg-shells after only 1–36 min for MW-HT, compared to several hours when using HT alone; a higher pH (between 9–11) and reaction time generate a high content of carbonate groups in the resulting HA lattice [53,59,60,61], but the high pressure in the synthesis system ensures a high internal and external porosity, as has been reported only in few papers [60]. Besides, a wide range of morphologies were obtained only using the two combined techniques for nano-hydroxyapatite synthesis. Furthermore, the association between the two techniques, HT and MW, was reported to improve the control of particle size, porosity, and morphology by a better monitoring of process parameters (time, temperature, and energy), with low energy consumption, low temperatures (less than 250 °C), and short time process cycles [51,52,53,54,55,56,57,58,59,60,61].

In this paper, we compared three categories of biomaterials obtained from natural sources, two represented by Bio-Oss^®^—bovine bone and Gen-OS^®^—porcine bone, bone grafts already used in dentistry with good clinical results [18,62,63,64], and the last one being biomimetic synthetic hydroxyapatite from egg-shells (HA1), synthesized by the microwave-assisted hydrothermal technique (HT-MW), after only two hours of treatment at 200 °C. The usage of this unconventional, hybrid synthesis method with microwave heating has already been reported in the literature with good results. The fact that a natural calcium source has been proposed as a starting material aims to bring added value to this work and proposes an alternative to the actual, expensive, commercial materials. Moreover, the paper is intended as an extensive comparative study between the synthetic material and the two bone grafts already used in dentistry, highlighting their chemical, structural, morphological, and biological resemblance.

## 2. Materials and Methods

### 2.1. Materials

Gen-Oss^®^ powder was purchased from Tecnoss Dental (Pianezza, Italy). It is a mixture of grinded cortical (20% wt.) and cancellous (80% wt.) porcine bone, obtained by low-temperature treatment (maximum 130 °C) in order to partially burn out the organic compound of bone, the version with grain sizes of 250–1000 µm.

Bio-Oss^®^ powder was purchased from Geistlich Pharma AG (Wolhusen, Switzerland). It is a chemically and thermally treated cancellous bovine bone. Hence, grinded bovine bone was first deproteinized by reaction with the strong alkali medium, and then calcined at 300 °C, the version available with 0.25–1 mm grain sizes.

Hydroxyapatite (HA) was synthesized from a natural calcium carbonate source—hen egg-shells.

#### 2.1.1. Calcium Oxide Precursor Preparation

Here, 100 g of hen eggshells were harvested from a local poultry and boiled for 4 h in water with 10 mL of H_2_O_2_, for complete removal of the organic part, and then washed with distilled water, dried in an oven at 60 °C, and ground for 15 min. The ground material was annealed in an electric oven, with a temperature rise rate of 10 °C/min, up to a temperature of 800 °C, bearing 3 h, and then slowly cooled to ambient temperature for 24 h.

#### 2.1.2. Hydroxyapatite Preparation

Here, 45 g of annealed egg-shell powder was dispersed in 200 mL of distilled water and further used as a Ca source. Then, 100 mL of aqueous solution of 38.5% of di-base ammonium phosphate [(NH_4_)_2_HPO_4_] was prepared and used as a phosphorus precursor. The two reagents were mixed under continuous magnetic stirring, by dripping di-base ammonium phosphate over a Ca source at an average speed of 2 mL/min, periodically adjusting the pH > 11. This value has been reported in the literature to favor the formation of rod-like hydroxyapatite [30]. The precipitate obtained was maturated in a microwave-assisted hydrothermal Teflon autoclave.

#### 2.1.3. Microwave-Assisted Hydrothermal Maturation of Hydroxyapatite Precipitate

The precipitate was introduced into a 50 cm^3^ Teflon vessel with an occupancy rate of 50% [59], being subjected to a hydrothermal-microwave heating treatment as follows: the temperature increased from room temperature to 200 °C at a rate of 35 °C/min, where it was maintained for 30 min, and then slowly decreased back to room temperature. Throughout the heating cycle, the MW energy supplied to the system varied in the first 5 min of treatment between 1.2 and 1.6 kW and, during the 30 min at maximum temperature, the range was <1.0 kW. The pressure in the system increased during the hydrothermal maturation in the first 40 min, from the initial input value of 16 bar at approximately 20 bar, remaining around this value also during the cooling period. The maturated precipitate was filtered and washed with distilled water until pH = 7, and then dried at 60 °C for 48 h, resulting in HA1 powder.

### 2.2. Characterisation Methods

X-ray diffraction (XRD) was performed using a PANalytical Empyrean Spectrometer (Malvern PANalytical, Bruno, The Netherlands), operating in a Bragg-Brentano configuration with Cu-Kα (λ = 1.5406 Å). The spectra were recorded at 100 < 2θ < 80° with a scan speed of 0.5°/min and a step size of 0.02°. Using the following empirical equation, Equation (1), the crystallinity degree for every powder diffraction pattern can be appreciated [46]:

(1) $$Xc=1-\frac{V112/300}{I\;300}$$

where I_300_ is the intensity of the reflection crystal plane (300) and *V*_112/300_ is the intensity of the difference between (112) and (300) reflections (which completely disappears in noncrystalline samples of hydroxyapatite) [65,66,67].

In order to calculate the average hydroxyapatite crystallite size of Bio-Oss, Gen-OS, and HA1 powders, the Rietveld method was applied, based on all X-ray diffraction peak profiles in the pattern, using HighScorePlus 3.0.e software and the pseudo-Voigt function for the profile refinement procedure [65,66,67]. The Scherer formula (2) was used to estimate the crystallite size only along the growth plane direction using a convolution of the Cauchy–Lorentz probability distribution that is marked in the XRD diagram by an increasing peak associated with a higher crystallinity degree of the powders [61]:

(2) $$r=\frac{K\lambda }{B\;\cos\theta }$$

where *r* = crystallite size [nm], *K* = 0.9 constant [61], *λ* = wavelength of monochromatic X-ray beam [nm] (*λ* KαCu = 0.15418 nm), *B* is the full-width of the peak at half-intensity of each crystalline plane reflection [rad], and *θ* is the exact diffraction angle [rad].

Fourier-transform infrared spectroscopy (FTIR) spectra were recorded in the wavenumber range of 4000–500 cm^−1^, in increments of 1.928 cm^−1^, using a Nicolet iS50R spectrometer (Thermo Fisher, Waltham, MA, USA), in attenuated total reflection mode (ATR). Each spectrum was collected at room temperature at a resolution of 4 cm^−1^, and 32 samples were scanned between 4000 and 440 cm^−1^. The obtained results were presented as the average of the 32 individual scanned samples for each Bio-Oss, GenOs, and HA1 powder and were compared with the theoretical available data [68,69,70,71].

A Quanta Inspect F scanning electron microscope (SEM) (Thermo Fisher, Eindhoven, the Netherlands), equipped with a field electron emission gun (FEG) and an EDS (energy-dispersive spectroscopy) detector, was used. The technical parameters were: acceleration voltage of 30 kV and point-to-point resolution of 1.2 nm.

A TECNAI F 30G2 SWIN transmission electron microscope (TEM) (Thermo Fisher, Eindhoven, the Netherlands) was used, with a 300 kV acceleration transmission with a Shottky electron emission, HRTEM point and line resolutions of 2 Å and 1.02 Å, respectively, 60x-1Mx magnification range, and a minimum diffraction angle of ±12°, equipped with an EDS probe. 

The metal contents of both HA1 powder and egg-shell raw material were determined by X-ray fluorescence spectra (XRF) using a Thermo Scientific ARL PERFORM’X Sequential spectrometer, which works under pressure in the He atmosphere, and the purchase was made according to the Thermo Scientific UniQuant soft, nonstandard method.

In vitro qualitative biocompatibility was performed on a GM0047 amniotic fluid stem cell line (AFSC), purchased from Coriell Institute (Kenton, NJ, USA) and cultivated at the Faculty of Veterinary Medicine, Department of Biochemistry (Bucharest, Romania). The cells were cultivated in Dulbecco’s modified Eagle’s medium (DMEM, Sigma-Aldrich, Missouri, MO, USA) supplemented with 10% fetal bovine serum and 1% antibiotics (penicillin and streptomycin) and changed twice a week. The AFSC cell culture was obtained at a final concentration of 5 µM and RED CMTPX was added as the cell trace fluorophore. The cells were treated with Bio-Oss, Gen-Os, and HA1 granular materials and incubated for 30 min, allowing the chromophore penetration into the cells. The viability and morphology of the AFSCs were appreciated after 5 days. The cell phenotype was evaluated by flow cytometry using specific markers such as SSEA-1, SSEA-4, TRA1–60, TRA 1–81, CD90, CD73, CD56, CD49E, CD44, CD31, CD105, and CD45. There was no modification in the cell phenotype after 5 days in the presence of the biomaterials [72,73]. After this period, in order to observe the cells’ fluorescence, the AFSC medium was washed with phosphate-buffered saline (PBS) (8.0 g/L of NaCl, 0.2 g/L of KCl, 1.42 g/L of Na_2_HPO_4_, and 0.24 g/L of KH_2_PO_4_, pH~7.4). The micrographs were made with a digital camera Olympus CKX 41, driven by CellSense Entry software (Olympus, Tokyo, Japan).

Two quantitative evaluations of the in vitro cellular bioactivity in contact with the three biomaterials were performed: Viability and Oxidative Stress Assessments (MTT and GSH-Glo Glutathione Assays). The GSH-Glo Assay is based on the conversion of a luciferin derivative (Luc-NT-luciferin dimer) coupled in the presence of glutathione (GSH), oxygen, enzymes (luciferase and glutation S transferase), and ATP. Adding the marker of luciferase Ultra-Glo Recombinant Luciferase is necessary to produce luminescence, which is proportional to the quantity of GSH produced in cells. GSH is the main thiol of animal cells involved in important metabolic mechanisms such as signaling biomolecules in redox reactions, the regulation of cell proliferation, and fibrogenesis, and its level is a measure of the antioxidative stress of cells [74]. AFSCs were seeded for 24 h, at a density of 3000 cells in 300 µL of DMEM supplemented with 10% fetal bovine serum and 1% antibiotics (penicillin and streptomycin/neomycin) in 96-well plates. After preparing the cell culture, the tested biomaterials (Bio-Oss, Gen-Os, and HA1) were put in contact and incubated for 72 h. The protocol involves the addition of 100 µL of 1X GSH-Glo Reagent and incubating it at 37 °C for 30 min, followed by adding 100 µL of Luciferin Detection Reagent for another 15 min, also incubated at 37 °C. Three independent wells of each sample were observed on a luminometer (Microplate Luminometer Centro LB 960, Berthold, Germany), after a good homogenization. The change in density, produced by solubilized formazan, was appreciated spectrophotometrically (TECAN Infinite M200, Männedorf, Switzerland) (Thermo Fischer Scientific, Waltham, MA, USA); the absorbance of solubilized formazan concentration is proportional to the metabolic activity of living cells in the culture. The human mesenchymal amniotic fluid stem cells (AFSCs) (Vybrant^®^MTT Cell Proliferation Assay Kit) were cultured in 96-well plates, with a seed density of 3000 cells/well, in the presence of analyzed Bio-Oss, Gen-Os, and HA1 powders, into DMEM medium (Sigma-Aldrich, Saint Luis, MI, USA) with the addition of 10% fetal bovine serum, 1% penicillin, and 1% streptomycin antibiotics (Sigma-Aldrich, Saint Luis, MI, USA), for 72 h. After the incubation period, 15 mL of MTT (12 mM) was added and kept for 4 h at 37 °C, and using a pipette, the solution of 1 mg of sodium dodecyl sulphate + 10 mL of HCl and 0.01 M was appended to the formazan crystals solubilization. The absorbance was measured after 1 h, in triplicate, using a spectrophotometer at 570 nm [75,76,77,78].

## 3. Results and Discussions

The synthesis strategy for the HT-MW maturation treatment was to control the process parameters in order to obtain a nanosize single-phase hydroxyapatite powder, with particles presenting mesopores. The usage of microwave radiation leads to a higher reaction speed, due to the polarization of water from the aqueous suspension [20]. Consequently, the treatment duration was reduced to only 30 min and the entire process took place with energy consumption savings. The maximum temperature of the process was settled at 200 °C in order to avoid the formation of secondary phosphate phases, as reported in the literature [19,20]. A homogeneous nanosize powder can be acquired by using the precipitation and hydrothermal synthesis methods [24,27], but taking care that the treatment time and temperature do not exceed the conditions for crystal growth velocity [20]. The rod-like morphology of the synthesized hydroxyapatite particles have been reported [52,53,54], even using the solvothermal-MW method, with different organic phosphate precursors [49,50,51,52], and the suspension pH seems to be the decisive parameter [57]. Therefore, the pH was carefully kept, during precipitate development, at a high basicity level over 11 by choosing a suitable regent. Adopting a high-pressure hydrothermal process (initial 16 bars) combined with gaseous reaction products, a high porosity of crystals was expected.

**Figure 1:**
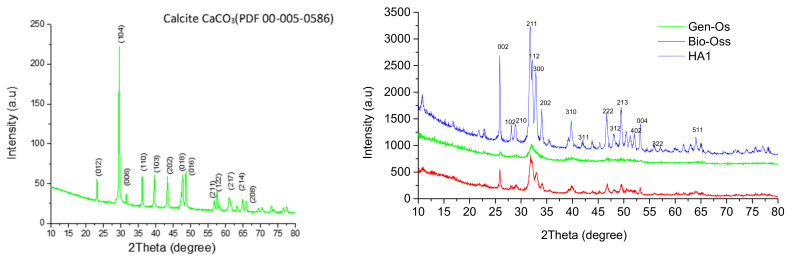
XRD plot for: (**left**) hen egg-shell powder before calcination; (**right**) HA1, Bio-Oss, and Gen-Os powders.

**Figure 2:**
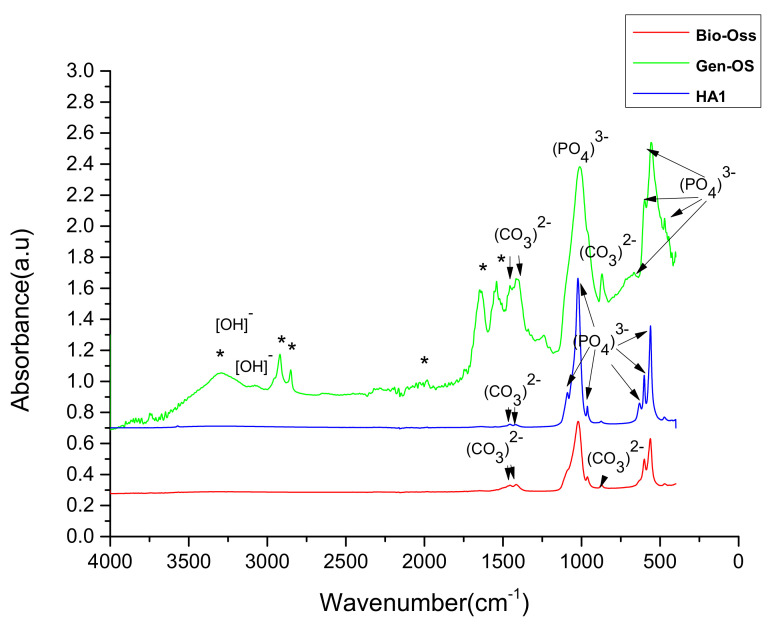
FTIR absorption spectra for xenograft Bio-Oss, Gen-Os, and HA1 powder: (*) absorption bands for protein functional groups.

Figure 1 (left) represents the XRD pattern of the natural raw material (hen egg-shells) used for hydroxyapatite synthesis, before calcination, while Figure 2 comparatively presents the diffraction patterns for all three apatite materials. The XRD pattern in Figure 1 was matched by the calcite (CaCO_3_) phase with a high degree of crystallinity evidenced by sharp diffraction peaks. The results are in good agreement with numerous references, which present egg-shells as a calcium carbonate source [24,25]. In addition, a calcium content of 96.38 wt.% was observed after XRF examination, made on egg-shells before calcination. 

According to the reference sheet PDF code 00-009-0432, the sample HA1 consists of 100% HA, and all characteristic crystalline planes were present with amplitude and diffraction angles (2θ) corresponding to a hexagonal symmetry hydroxyapatite pattern, as seen in Figure 1 (right, blue). No other secondary phases were present in the HA1 sample. For Bio-Oss^®^ (Figure 1 right, red), the following main characteristic crystalline planes of HA were distinguished: (211) with a maximum amplitude (100%) at 2θ of 31.77°, followed by (112) and (300) of 60% at 2θ = 32.19° and 32.90°, respectively, (002) at 25.87° (40%), (310) at 2θ of 39.80° and (222) at 46.79°, (213) (18%) to 49.46°, and (004) to 53.14°. Gen-Os (Figure 1 right, green) proved to be the least crystallized sample of the three, as only the crystalline phase planes (002), (211), (112), and (310) of HA were identified. Moreover, Gen-Os registered a much smaller intensity and broad X-ray scattering profile at low diffraction angles, compared with the other two samples. As Gen-Os originated from porcine bone after thermal treatment at low temperature, the decrease in crystallinity can be attributed to the possible small numbers of remaining organic components, an hypothesis that was later confirmed by FTIR analysis (Figure 2). Compared to Gen-Os, Bio-Oss was better-crystallized, but the exhibited peaks corresponding to the crystalline planes of HA were smaller in intensity compared to the HA1 powder XRD pattern. From XRD patterns, applying Equation (1), the crystallinity degree was calculated, proving the highest crystallinity for HA1 (XC = 0.92), followed by Bio-Oss (XC = 0.56) and Gen-Os XC = 0.28 [46].

To determine the crystallinity degree and crystallite sizes of hydroxyapatite corresponding to the three samples of bone substitutes, the Rietveld method and Scherer equation were used [65,66,67]. To calculate the average size of crystallites, the Rietveld method is more precise compared with the Scherer equation, because it takes into consideration all peak separations or the total integrated intensity of groups of overlapping peaks from the diffraction plot, using a pseudo-Voigt function for a better matching profile of X-ray diffraction peaks. Using each of the three XRD patterns with the application of the Rietveld method, the crystallite average size of HA in the HA1 sample was the largest of the three samples (21.62 nm), almost double compared to Bio-Oss and almost triple compared to Gen-Os (Table 1), as already suggested by the small width of the HA1 XRD peaks presented in Figure 1 [65,66,67].

Table 2 shows the calculated crystallite sizes for [002], [211], and [300] planes, having the highest diffraction peaks in each plot for the three samples HA1, Gen-Os, and Bio-Oss. Even though the (211) plane is associated with the highest-intensity peak for all samples, its corresponding crystallite sizes are not largest. It can be observed that the biggest crystallite growth occurs under the (002) plane for samples HA1 and Bio-Oss, of 23.44 nm and 43.73 nm, respectively, which was found in the literature to be typical for natural bone, where the *c* axes are the growth direction for collagen fibrils [68]. The Gen-Os sample shows the smallest crystallite sizes, including under the [002] direction, explained by the lowest amplitude of all diffraction peaks being registered for this sample (Figure 2 green), perhaps because small hydroxyapatite crystals are shielded by the wrapping of proteins. The results obtained through both calculation techniques are comparable and present the same order for increasing the mean crystallite size of Gen-Os < Bio-Oss < HA1.

The FTIR spectra in Figure 2 are attributed to HA1 (blue line), Bio-Oss (red line), and Gen-Os (green line) powders. Symmetric vibration bending or stretching of the absorption bands can be observed for the C-O bond at wavenumbers of 1454, 1420, and 874 cm^−1^, which can be attributed to CO_3_^2−^ (carbonate ions type B) that substitutes PO_4_^3−^ in the HA lattice. The PO_4_^3−^ groups absorption bands identified at 472 cm^−1^ are characteristic of the ν _2_ inclination of the O-P-O bond, the high-amplitude bands from 572 cm^−1^, 601 cm^−1^, and 963 cm^−1^ refer to the symmetric and asymmetric deformation modes of ν _4_ O-OP, while the intense absorption bands in the range of 1040–1090 cm^−1^ correspond to the ν _3_ P-O [69,70,71].

The absorption bands from 3356 cm^−1^ marked with arrow refer to the bending oscillation modes of [OH]^−^ structurally bound in the HA lattice that is present for Gen-Os but is overlapped with absorption bands characteristic of proteins or lipid functional groups, as well as physically adsorbed water. The absence of absorption bands for O-H is mentioned in different papers as a characteristic of biological apatite, explainable for xenografts such as Bio-Oss. In addition, for specimen HA1, the [OH] group absorption band in the range of 3600–3200 cm^−1^ is missing, which could prove that the hydroxyapatite synthesis from the natural source (egg-shells) leads to an analogous composition to bio-apatite from Bio-Oss. The presence of the [CO_3_^2−^] group in HA1 that substitutes [PO_4_^3−^] groups from the HA lattice, with absorption peaks reported at wave numbers of 1454 cm^−1^, 1420 cm^−1^, and 874 cm^−1^ as in natural bone, could be proof of compositional similarity. The strong absorption band at 874 cm^−1^ was assigned to the presence of CO_3_^2−^ involved in B-type PO_4_^3−^ substitution (ν_2[B]_), and the absence of a lower intensity for the absorption band at 880 cm^−1^ attributed to A-type carbonate substitution (ν_2[A]_ in OH^−^ position) together with the missing band at around 3570 cm^−1^ for the stretching mode of OH^−^ could be another piece of evidence. There are papers that presented methods to appreciate the content of CO_3_^2−^ that substitutes PO_4_^3−^ in bio-apatite (C/P), by measuring the amplitude of the absorption band at 1415–1420 cm^−1^ (as CO_3_^2−^quantity) and at 1011–1042 cm^−1^ corresponding to PO_4_^3−^ quantity [69,70,71].

According to this method, the C/P ratio for HA1 was 0.028, that for BioOss was 0.125, and the highest content of carbonate for GenOs was 0.574, the latter containing a certain quantity of denatured collagen but without any confusion, and the characteristic absorption band for carbonyl (CO) group is placed at 1455 cm^−1^ [69,70,71].

Besides, absorption peaks at 2920 cm^−1^ (reported at 2923 cm^−1^) and 2851 cm^−1^ are assigned to the stretching vibrations of CH, and wavenumbers of 1230, 1541, and 1648 cm^−1^ are absorption bands for C-H bonds in (CH2) and (CH3), and C-N, N-H, and C=O, respectively, for amide I, amide II, and amide III (reported absorption bands with maximum amplitude at wavenumbers of 1659–1555 cm^−1^), and 1011 cm^−1^ (reported at 1035 and 1079 cm^−1^) is assigned for bond vibrations ν(C–O) and ν(C–O–C), all of which have been reported for collagen type I [68].

**Figure 3:**
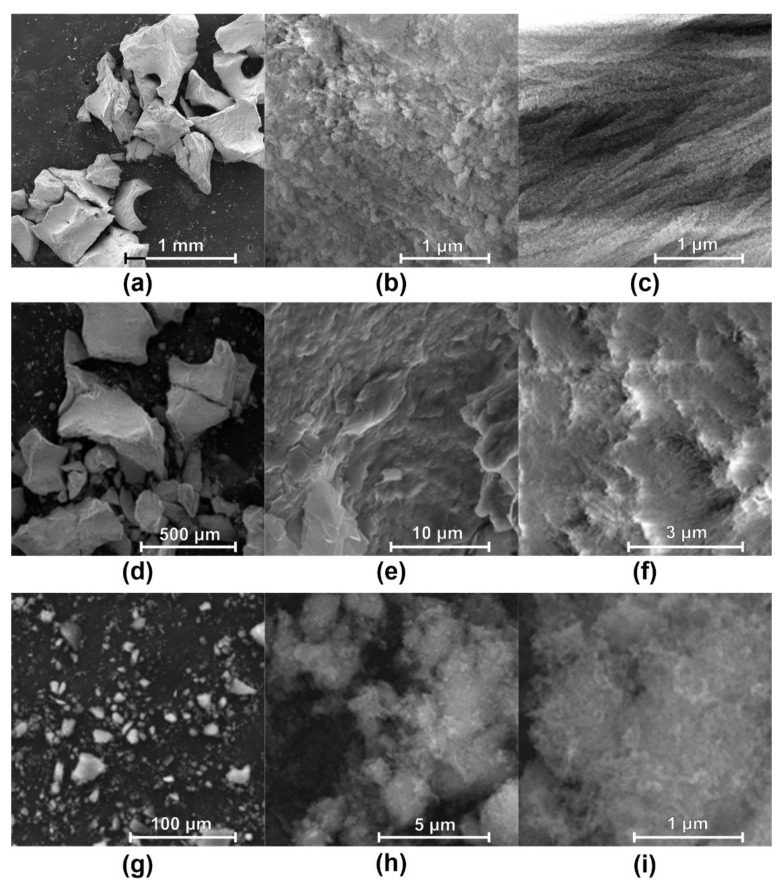
SEM images for: (**a**–**c**) Gen-Os (200×, 10,000×, and 40,000×); (**d**–**f**) Bio-Oss (200×, 10,000×, and 40,000×); (**g**–**i**) HA1 synthesized from egg-shells (1000×, 20,000×, and 100,000×).

The shape of the grains for the two xenografts resembles fragments of cancellous or cortical natural bone [18,62,63], while for the HA1 sample, the grains are very small and of prismatic shape (Figure 3g). At higher magnifications, the SEM images of the two xenografts show the characteristic morphology of the extracellular matrix in the bone tissue, with a very dense structure (Figure 3b,f), HA platelets crystallized in parallel planes, with nanometric width and 59–62 nm diameter, along with collagen fibers with hydroxyapatite mineralized on surface (Figure 3 c). For Gen-Os and Bio-Oss, the structure is caused by organic–inorganic nanocomposite biogenesis. Contrariwise, the HA1 sample SEM images indicate a soft and fluffy structure characteristic of the crystallization from precipitate, ultra-fine nanoparticle aggregate structures (5.44–7.69 nm), and no visible intergranular limits (Figure 3h,i) [74]. In a saturated solution, heterogeneous nucleation always takes place earlier than the homogeneous one, due to lower nucleus-free energy on foreign bodies [75].

**Figure 4:**
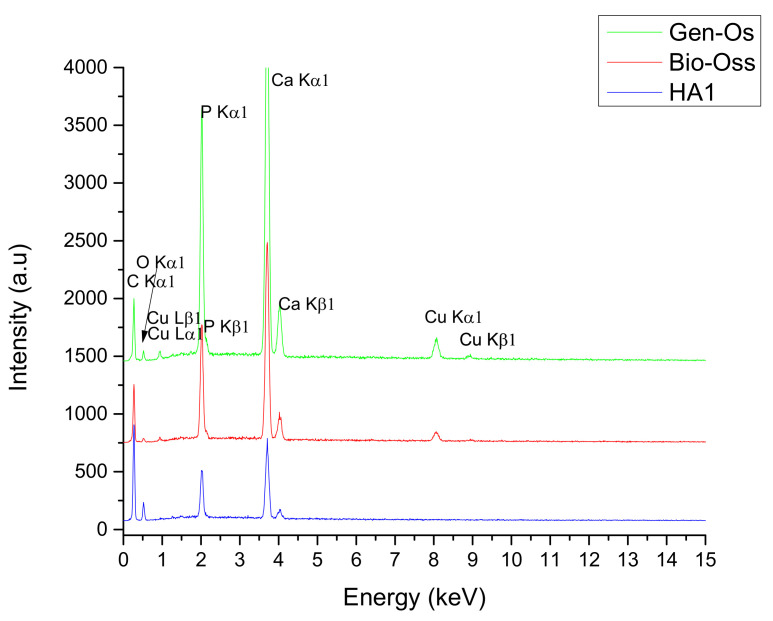
EDS spectra for: (red) Bio-Oss; (green) Gen-Os; (blue) HA1 powders.

The chemical elements identified in each sample are presented in the EDS spectra (Figure 4), and the elemental composition of HA1 and egg-shells before annealing powders can be observed in the XRF results (Table 3). The identified elements in the Bio-Oss and Gen-Os sample (C, P, O, and Ca) can be assumed to form a single phase of carbonated apatite with molar ratios of Ca:P~1.65 and 1.60, respectively. This is a well-known deviation from a hydroxyapatite unit cell stoichiometry (Ca/P = 1.67), which is in good correlation with the FTIR and XRD analysis results, where the nature of the sample source and the data is reported in the literature.

Comparing the results of the elemental analyses from the EDS dispersion spectra, which have only a qualitative value, the presence of copper in the two xenograft samples is highlighted, in a higher content at the Gen-Os sample. In addition, an evident carbon content is observed in all three samples, which may arise from the carbonate groups in Bio-Oss and HA1, but may have an organic and inorganic nature in Gen-Os. The amplitude peak corresponding to the C element, found at around 0.3 eV, could be attributed to graphite conductive tape, too. None of the EDS spectra for any of the three samples mark the presence of trace elements, as reported in the literature [18,36,37,38].

Studies in the literature have reported biological apatite derived from bone products with a Ca/P ratio between 1.50 and 1.85, strongly dependent on bone species and the age factor [36]. The responsible factors for this stoichiometry deviation are the cationic and anionic substitutions of calcium, phosphate, or hydroxyl groups from the hydroxyapatite lattice with trace elements and carbonate or silicate groups, respectively [18]. Using the EDS spectrum for HA1 (Figure 4), the calculated ratio of Ca/P = 1.69 ± 0.1 exceeds the ratio of these two elements in stoichiometric hydroxyapatite, with the same ratio being reported in the literature for synthesized hydroxyapatite but also for biologic apatite provided from biogenic sources.

XRF analysis on the synthesized hydroxyapatite sample (HA1) and unannealed egg- shell powder used as a source for this synthesis (Table 3) showed the presence of important trace elements in the egg-shell source, kept during all treatments, and also found in a smaller amount in HA1 nano-powder. Hence, the Na content decreased from 1.82 ± 0.07% wt. in egg-shells to 0.63 ± 0.23% wt. in HA1, the Mg from 0.98 ± 0.05% to 0.59 ± 0.04%, and so on. Such elements are known to be of great importance in the biocompatibility of natural hydroxyapatite and beneficial for the synthesized HA1. Many trace elements have been reported to substitute Ca ions of biologic HA, such as 0.9%wt Na^+^ and 0.5%wt Mg^2+^; Sr ^2+^ < 0.1%. PO_4_^3−^ groups are also usually substituted by around 4–6% CO_3_^2−^ [36]. In fact, bone mineral is a calcium-deficient apatite, where a Ca:P ratio of 1.67 is only the theoretical value for pure hydroxyapatite [36].

**Figure 5:**
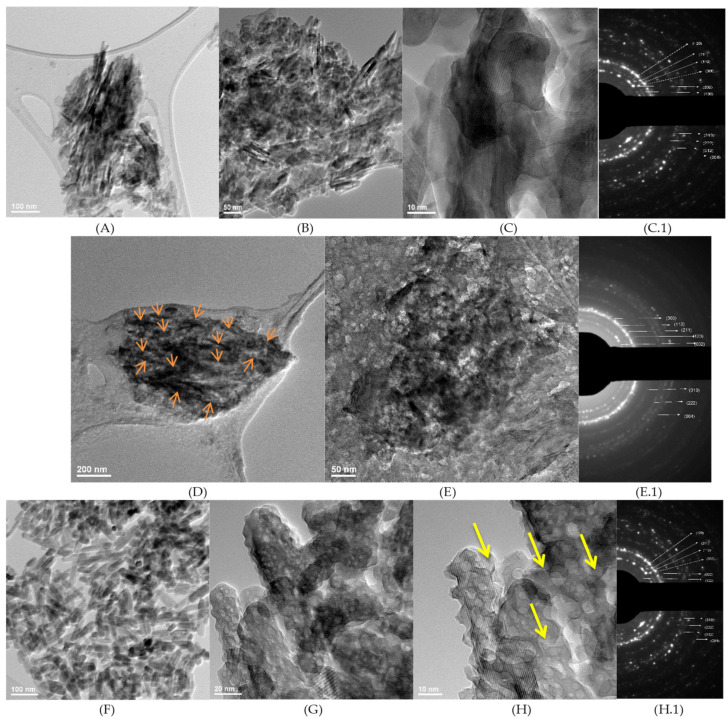
TEM images and SAED patterns for: (**A**–**C**) Bio-Oss, (**C.1**) SAED pattern Bio-Oss; (**D**,**E**) Gen-Os (rod-like particles- red arrows), (**E.1**) SAED pattern Gen-Os; (**F**–**H**) HA1 (intra-particle pores-yellow arrows), (**H.1**) SAED pattern HA1.

**Figure 6:**
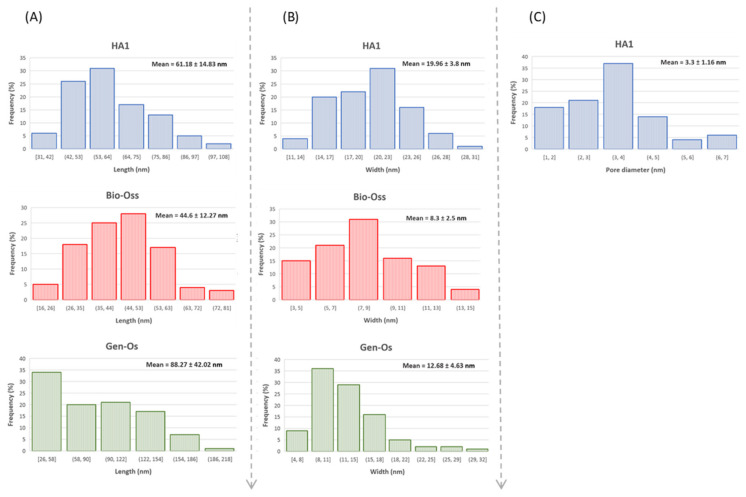
HA1 (blue), Bio-Oss (red), Gen-Os (green) particles length distribution (**A**) and width distribution (**B**); internal pores size distribution for HA1 sample (**C**).

TEM images for Bio-Oss, Gen-Os, and HA1 samples can be seen in Figure 5A–H. At smaller magnification, crystalline aggregates composed of polyhedral and rod-like hydroxyapatite particles are observed for Bio-Oss and HA1 samples (Figure 5D orange arrows and Figure 5G), while for the Gen-Os sample, the rod-like particles are faded by the presence of remnant collagenous components. The rod-like morphology is confirmed at the higher magnification (Figure 5B,G), resembling both Bio-Oss and HA1 samples. However, a higher surface roughness and numerous internal pores can be observed in the HA1 sample (Figure 5C,H, yellow arrows). Figure 6A,B show Bio-Oss particle mean lengths and diameters of 44.6 nm and 8.3 nm, respectively. Larger particles were measured for HA1, with a mean diameter of 19.96 nm and mean length of 61.18 nm, and the crystallites were elongated in the [002] growth direction under the *c* axis, as also proven by XRD analysis results (Figure 1 right). The diffraction rings presented in the SAED patterns are similar for HA1, Bio-Oss, and Gen-Os (Figure 5C.1,E.1,H.1), for each crystalline plane identified, proving the polycrystalline hydroxyapatite as the main component of the three samples. The ring associated with the (0 0 2) plane is present in all SAED patterns, which is characteristic to natural bone due to the collagen fibrils, and is evidence of the compositional similarity between the three samples [36]. The (1 1 2), (2 1 1), and (3 0 0) planes form three rings that overlap for all samples, appearing brighter. The diffuse light in the SAED pattern marks the presence of an amorphous phase in Gen-OS. 

For the Gen-Os, a sample of partially deproteinized porcine bone, the TEM images show the same polyhedral and nano-rod-like morphologies, with a mean length of 88.27 nm and mean diameter of 12.68 nm, larger than Bio-Oss. A possible explanation for these findings can be the reminiscence of natural bone proteins under the mineral structure, already proved by FTIR and SEM analysis. However, the sizes of Gen-Os particles are longer than those of HA1 (Figure 6A). Studies in the literature have reported bone hydroxyapatite crystals sizes to be 30–50 nm in length and 15–30 nm in width [36]. The width distribution of comparative particles is observed in Figure 6 B. In addition, the thinnest hydroxyapatite rod-like particles are contained by the Bio-Oss specimen (Figure 6B red), with the measured particle diameters for HA1 being of comparable size as those of Gen-Os.

The obtained results are in good agreement with previous literature studies, which have associated the morphology of synthetic HA with the hydrothermal conditions [51,52]. Hence, when treated at 150 °C for 24 h, needle-like structures are obtained [55], while for 72 h aging, the observed morphology is rod-like [51,52,59]. As could be seen in TEM, the micrographs associated with the HA1 sample (Figure 5G,H), the nano-rod-like crystals have high rough surfaces created by hydrothermal MW-assisted treatment of the HA precipitate without any templates. The rounded pores are irregularly distributed (Figure 5H, yellow arrow) and present on both the particle’s surface, as well as between grains, having an average size of 3.3 nm (Figure 6C) [60]. Comparing the TEM image of Bio-Oss in Figure 5C with the HA1 sample (Figure 5H), an obvious similar roughness of the particle’s surfaces could be seen, even if their synthesis history is different. Nevertheless, the sample HA1 has a very high internal porosity compared with Bio-Oss (Figure 5C,H yellow arrow).

**Figure 7:**
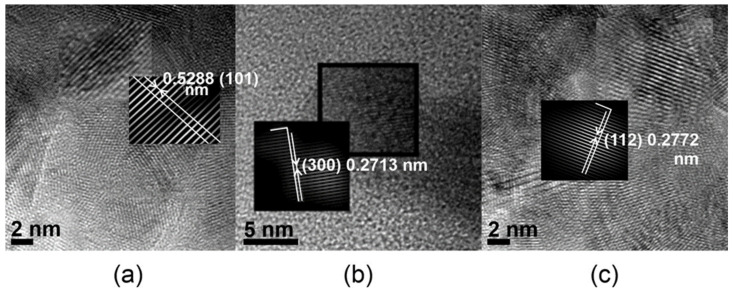
HRTEM images for specimens: (**a**) Bio-Oss (inset, inverse fast Fourier transform); (**b**) Gen-Os (inset, inverse fast Fourier transform); (**c**) HA1 (inset, inverse fast Fourier transform).

High-resolution transmission electron microscopy (HRTEM) images for every three specimens are presented in Figure 7a–c. This investigation allows the measurement of crystallites sizes on the selected area, as well as determining the *d*-spacing of the family of parallel planes belonging to a crystallite oriented under a certain direction in the polycrystalline particle, by measuring the distance between crystallite atom planes. In Figure 7a (inset), the d-spacing of 5.2881 Å corresponds to (101) Miller’s indices of hydroxyapatite crystals in the Bio-Oss sample, 2.7131 Å is the d-spacing for parallel planes in direction (300) in Gen-Os (Figure 7b), while the crystalline plane (112) has a *d*-spacing of 2.7613 Å for HA1 (Figure 7c). Some differences between the crystallite mean size determined from HRTEM and those calculated by Rietveld equations with XRD data were observed. A possible explanation could be the fact that HRTEM images show only part of a particle, with few crystallites grown under some crystalline planes, while the Rietveld method provides the mean size of crystallites, taking into consideration all crystalline planes of the sample.

**Figure 8:**
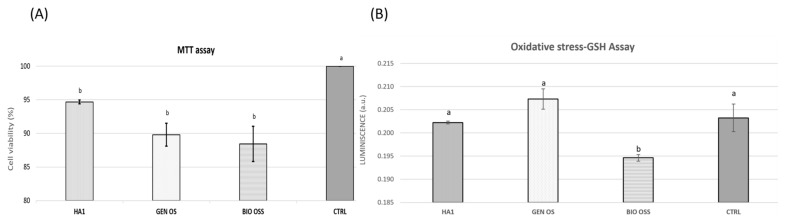
(**A**) MTT assay results (after 72 h incubation) and (**B**) GSH assay showing the oxidative stress of AFSCs cultured in the presence of HA1, Gen-Os, Bio-Oss powders, and CTRL sample (only cells); the results are presented as the mean ± S.D. of 3 replicates; different letters indicate significant differences between each sample; *p* < 0.05/n (n = 6)-based ANOVA statistical analysis, followed by a two-tailed *t*-test with Bonferroni post hoc correction (**A**).

The percentage of viable cells after the 72 h incubation period (Figure 8A) was calculated by taking the ratio between the absorbance recorded on cell cultures in the presence of biomaterial powders and that of the control sample (CTRL) [76,77,78]. The metabolic activity of AFSCs in the presence of HA1 powder proves to be higher than the cell viability in the presence of the two xenografts, with the lowest viability in the series being registered in the presence of Bio-Oss powder. However, the one-way analysis of variance (ANOVA), followed by a two-tailed *t*-test with Bonferroni post-hoc correction results, showed that the differences between Bio-Oss, Gen-Os, and HA1 samples are not statistically significant. Yet, there is a statistically significant difference between the cellular viability registered in their presence and the control sample, which contains only AFSCs. Hence, even though the analyzed powders are inhibited in a small proportion, the metabolic activity of the AFSCs, HA1 sample proves similar enough with the two commercial xenografts already used as a bone substitute in dentistry. Consequently, the already mentioned morphological and structural differences between Bio-Oss, Gen-Os, and HA1 do not greatly influence their biocompatibility.

In the GSH assay, the cytotoxic effect translates into an increased oxidative stress generated on the cells in the presence of HA1, Gen-Os, or Bio-Oss samples, and for the control sample, consisting of cells only. An intense AFSC bioactivity leads to more GSH enzymes coupled to fight against oxidative species, forming glutathione disulfide (GSSG). The GSH/GSSG molar ratio represents a powerful index of oxidative stress and disease risk and can be determined by numerous analytical methods, including UV-Vis spectrophotometry. The measured luminescence is proportional to the amount of GSH involved in the antioxidative stress, and as shown in Figure 8B, the most stress-free AFSCs are those in contact with Bio-Oss, with the HA1 sample coming next. The CTRL sample is placed before Gen-Os, which manifests the strongest oxidative stress on the cell line cultured, all incubated for 24 h. However, data analysis results showed that the differences between CTRL, Gen-Os, and HA1 samples are not statistically significant. Yet, there is a statistically significant difference between the oxidative stress registered in their presence and the Bio-Oss sample. Even though the Gen-Os and HA1 powders seem to cause an increase in GSH amount, unlike Bio-Oss, their cytotoxic effect is nonsignificant compared to cells only (CTRL) and can be considered biocompatible, similar to MTT analysis results. The GSH test proves once more the biological resemblance between the HA1 sample and Gen-Os xenograft, but also highlights the atypical behavior of Bio-Oss. Analyzing the morphological and structural characteristics of Bio-Oss, compared with Gen-Os and HA1, a prospective correlation with their biological properties arises: smaller particles manage to diminish the oxidative stress level by restoring the balance between the formation of reactive oxygen species (ROS) in cells and the capability of the cells to clear these free radicals; the smallest hydroxyapatite rod-like particles were observed for the Bio-Oss sample, while the measured particles for HA1 were comparable in size with Gen-Os.

**Figure 9:**
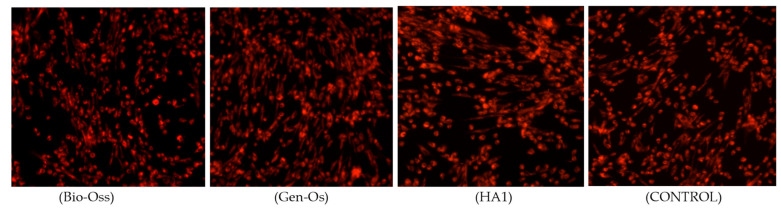
Fluorescent microscopy images of Bio-Oss, Gen-Os, HA1, and CONTROL colored with CMTPX fluorophore.

The fluorometric microculture cytotoxicity assay (FMCA) is an in vitro nonclonogenic-based cell viability assessment used for the cytotoxic and cytostatic measurement effect of different compounds or biomaterials, after a short time of incubation. The assay is based on fluorescein diacetate (FDA) hydrolysis by esterase in cells, keeping intact plasma membranes [79]. AFSCs were seeded under the described protocol (control cells only) and in contact with Bio-Oss, Gen-Oss, and HA1 samples, which could be observed as viable at the fluorescence microscope, after 72 h of incubation, with cells absorbing CMTPX fluorophores added in the cytoplasm (Figure 9). Fluorescence microscopy images show that AFSCs are viable and preserve their initial morphology, with homogenous sizes and density distributions in the culture well plates. No fragmented or dead cells could be identified on the background in the presence of xenografts and HA1 powders, as well as without any biomaterials in contact. In addition, from the images, the formation of filopodia actin-rich protrusions attached by many cells could be seen. As they are usually involved in numerous cellular processes, including cell migration, wound healing, adhesion to the extracellular matrix, and guidance toward chemoattractants, it demonstrates that seeded AFSCs exhibit a good comparable bioactivity for all samples analyzed [79].

## 4. Conclusions

Nano-hydroxyapatite (HA1) was synthesized from egg-shells by the microwave-assisted hydrothermal method (HTMW). From this hybrid, through an unconventional genesis route, a crystalline, homogenous in dimension, rod-like hydroxyapatite was obtained, after only several minutes, compared to various literature studies that reported several hours when using a hydrothermal technique alone. The obtained material demonstrates a mimetic composition, morphology, and structure with the commercial xenografts Bio-Oss^®^ and Gen-OS^®^. The fact that the HA1 sample, unlike the two xenografts, proved to have a very high meso-porosity was noticeable. This could be associated with an improved biomolecule adhesion and a potential increased osteoconductivity, and could be the cause for the good results of this sample at all in vitro cytotoxicity assays. Moreover, HA1 can be utilized in granular form, as well as xenografts, with better bioactivity and osteoconductivity than the hydroxyapatite-based scaffolds. The HA1 powder thus synthesized has a high potential for applications in bone substitution, teeth fillers, or drug delivery systems.

## Figures and Tables

**Table 1:** Crystallite average size for HA1, Gen-Os, and Bio-Oss by Rietveld method.

Sample	Crystallite Average Size (nm)	Standard Deviation Value
Bio-Oss	12.65	1.45
Gen OS	7.52	0.89
HA1	21.62	1.89

**Table 2:** Crystallite size by Scherer equation for the highest crystalline planes.

Sample	FWHM	2 θ (°)	Crystalline Direction	Crystallite Size (nm)	Mean Crystallite Size (nm)
Bio-Oss	0.347	25.88	[002]	23.44	14.69
	0.856	31.86	[211]	9.64	
	0.753	32.93	[300]	10.99	
Gen-Os	3.874	25.88	[002]	2.10	5.45
	1.351	31.99	[211]	6.13	
	1.017	33.10	[300]	8.14	
HA1	0.186	25.78	[002]	43.73	23.83
	0.752	31.87	[211]	10.98	
	0.447	33.10	[300]	18.53	

**Table 3:** Main elemental constituents of HA1 powder and egg-shells before annealing by XRF.

Identified Element	Egg-Shell before Calcination		HA1	
	(% wt.)	Est. Error (%)	(% wt.)	Est. Error (%)
Ca	96.38	0.09	69.44	0.23
Na	1.82	0.07	0.627	0.23
Mg	0.980	0.049	0.594	0.044
P_x_	0.334	0.017	29.14	0.030
S_x_	0.233	0.012	0.0540	0.0027
K	0.0669	0.0033	-	-
Sr	0.0626	0.0031	0.0381	0.0019
Si	0.0420	0.0027	0.0400	0.0049
Al	0.0185	0.0055	-	-
Cl	0.0163	0.0009	0.0134	0.0022
Fe	0.0141	0.0016	0.0092	0.0027

## Data Availability

Not applicable.
